# The prognostic value and molecular properties of tertiary lymphoid structures in oesophageal squamous cell carcinoma

**DOI:** 10.1002/ctm2.1074

**Published:** 2022-10-17

**Authors:** Yihong Ling, Jian Zhong, Zelin Weng, Guangrong Lin, Caixia Liu, Chuqing Pan, Hong Yang, Xiaolong Wei, Xiuying Xie, Xiaoli Wei, Huizhong Zhang, Geng Wang, Jianhua Fu, Jing Wen

**Affiliations:** ^1^ State Key Laboratory of Oncology in South China Collaborative Innovation Center for Cancer Medicine Sun Yat‐sen University Cancer Center Guangzhou China; ^2^ Department of Pathology Sun Yat‐sen University Cancer Center Guangzhou China; ^3^ Department of Thoracic Oncology Sun Yat‐sen University Cancer Center Guangzhou China; ^4^ Guangdong Esophageal Cancer Institute Guangzhou China; ^5^ Yinhe Hangtian Internet Technology Company Limited Beijing China; ^6^ Department of Preventive Medicine Shantou University Medical College Shantou China; ^7^ Department of Pathology Cancer Hospital of Shantou University Medical College Shantou China; ^8^ Department of Thoracic Surgery Cancer Hospital of Shantou University Medical College Shantou China

**Keywords:** deep learning, digital spatial profiling, oesophageal squamous cell carcinoma, prognosis, tertiary lymphoid structure

## Abstract

**Background:**

Tertiary lymphoid structures (TLSs) play key roles in tumour adaptive immunity. However, the prognostic value and molecular properties of TLSs in oesophageal squamous cell carcinoma (ESCC) patients have not been studied.

**Methods:**

The prognostic values of the presence and maturation status of tumour‐associated TLSs were determined in 394 and 256 ESCC patients from Sun Yat‐sen University Cancer Center (Centre A) and the Cancer Hospital of Shantou University Medical College (Centre B), respectively. A deep‐learning (DL) TLS classifier was established with haematoxylin and eosin (H&E)‐stained slides using an inception‐resnet‐v2 neural network. Digital spatial profiling was performed to determine the cellular and molecular properties of TLSs in ESCC tissues.

**Results:**

TLSs were observed in 73.1% of ESCCs from Centre A via pathological examination of H&E‐stained primary tumour slides, among which 42.9% were TLS‐mature and 30.2% were TLS‐immature tumours. The established DL TLS classifier yielded favourable sensitivities and specificities for patient TLS identification and maturation evaluation, with which 55.1%, 39.5% and 5.5% of ESCCs from Centre B were identified as TLS‐mature, TLS‐immature and TLS‐negative tumours. Multivariate analyses proved that the presence of mature TLSs was an independent prognostic factor in both the Centre A and Centre B cohorts (*p* < .05). Increased proportions of proliferative B, plasma and CD4+ T helper (Th) cells and increased B memory and Th17 signatures were observed in mature TLSs compared to immature ones. Intratumoural CD8+ T infiltration was increased in TLS‐mature ESCC tissues compared to mature TLS‐absent tissues. The combination of mature TLS presence and high CD8+ T infiltration was associated with the best survival in ESCC patients.

**Conclusions:**

Mature TLSs improve the prognosis of ESCC patients who underwent complete resection. The use of the DL TLS classifier would facilitate precise and efficient evaluation of TLS maturation status and offer a novel probability of ESCC treatment individualization.

## Graphical abstract

1. Presence of mature (tertiary lymphoid structures) TLSs was a promising prognostic factor for oesophageal squamous cell carcinoma patients who underwent complete resection.
2. A deep‐learning TLS classifier model for TLS identification and maturation evaluation was established to facilitate routine TLS evaluation and stratify prognosis in slide diagnosis.
3. Mature TLSs were characterized by increased proliferative B, plasma and CD4+ Th cells, and B memory and Th17 signatures

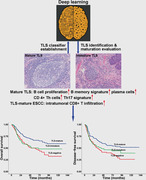

## 1 INTRODUCTION

Oesophageal cancer is one of the most aggressive malignancies of the gastrointestinal tract.^1^ As a major pathological type of oesophageal cancer, oesophageal squamous cell carcinoma (ESCC) ranks as the third most common malignancy in China and accounts for more than half of the global burden.^2^ Tumours, including ESCC, are increasingly recognised as complex organs that include a repertoire of recruited immune cells that contribute to the tumour microenvironment (TME) with cancer cells.^3^ These immune cells have been involved in each step of tumour development and are related to the prognoses of tumour patients.^4^

Tumour‐infiltrating T cells, especially CD8+ T cells, which are the primary mediators of anti‐tumour cellular immunity, have been a central focus of immunotherapy for the treatment of cancer^5^ and contributed positively to anti‐tumour immunity in different types of cancers, including ESCC.^6^, ^7^ However, the role of tumour‐infiltrating B cells, which provide humoral immunity against tumour cells, is less explored.^8^, ^9^ B cells in the TME are often organised into tumour‐associated tertiary lymphoid structures (TLSs). TLSs are ectopic lymphoid aggregates that develop in non‐lymphoid tissues at sites of chronic inflammation, including tumours, and are comprised of diverse immune cells, including but not limited to B and T cells. TLSs have been identified as prognostic factors in various types of tumours.^10^, ^11^ However, the prognostic value and molecular profiles of TLSs in ESCC have not been clarified.

In this study, we evaluated the prognostic value of TLSs in ESCC patients who underwent complete resection. A deep‐learning (DL) TLS classifier was developed to facilitate a more precise and efficient evaluation for TLS existence and maturation status based on haematoxylin and eosin (H&E)‐stained slides. The cellular and molecular properties of TLSs and tumours in ESCCs with different TLS status were examined using digital spatial profiling (DSP).

## 2 RESULTS

### 2.1 Patient characteristics

A total of 650 completely surgically resected ESCC patients were recruited, including 394 from Sun Yat‐sen University Cancer Center (Centre A) and 256 from the Cancer Hospital of Shantou University Medical College (Centre B) (Table 1). Patients were followed up with a median time of 68.57 months (interquartile range: 49.23–96.43 months) for the Centre A cohort and 52.17 months (interquartile range: 47.01–67.08 months) for the Centre B cohort. There were 6 (1.5%), 58 (14.7%) and 3 (0.8%) patients in the Centre A cohort and 38 (14.8%), 50 (19.5%) and 30 (11.7%) patients in the Centre B cohort receiving adjuvant radiotherapy, chemotherapy and chemoradiotherapy, respectively.

**TABLE 1:** Clinicopathological characteristics of TLS‐mature, TLS‐immature and TLS‐negative ESCC patients in Centre A cohort and Centre B cohort

	Centre A cohort					Centre B cohort				
Variable	Cases	TLS‐negative (*N* = 109)	TLS‐immature (*N* = 116)	TLS‐mature (*N* = 169)	*p* Value^b^	Cases	TLS‐negative (*N* = 14)	TLS‐immature (*N* = 101)	TLS‐mature (*N* = 141)	*p* Value^b^
Age (years)					.291					.028
≤60^a^	201	63 (15.4)	54 (26.9)	84 (41.8)		116	12 (10.3)	42 (36.2)	62 (53.4)	
>60	193	46 (23.8)	62 (32.1)	85 (44.0)		140	2 (1.4)	59 (42.1)	79 (56.4)	
Gender					.149					.914
Male	312	94 (30.1)	85 (27.2)	133 (42.6)		195	10 (5.1)	78 (40.0)	107 (54.9)	
Female	82	15 (18.3)	31 (37.8)	36 (43.9)		61	4 (6.6)	23 (37.7)	34 (55.7)	
Tumour location					.491					.477
Upper	56	15 (26.8)	17 (30.4)	24 (42.9)		46	3 (6.5)	21 (45.7)	22 (47.8)	
Middle	231	57 (24.7)	69 (29.8)	105 (45.5)		162	7 (4.3)	57 (35.2)	98 (60.5)	
Lower	107	37 (34.6)	30 (28.0)	40 (37.4)		48	4 (8.3)	23 (47.9)	21 (43.8)	
Tumour differentiation					.995					.757
Poor	113	30 (26.5)	34 (30.1)	49 (43.4)		50	1 (2.0)	21 (39.5)	28 (56.0)	
Moderate	243	69 (28.4)	71 (29.2)	103 (42.4)		106	5 (4.7)	38 (35.8)	63 (59.4)	
Well	38	10 (26.3)	11 (28.9)	17 (44.7)		100	8 (8.0)	42 (42.0)	50 (50.0)	
Number of retrieved LNs					.291					.914
<15	24	10 (41.7)	8 (33.3)	6 (25.0)		11	0 (0.0)	4 (36.4)	7 (63.6)	
≥15	370	99 (26.8)	108 (29.2)	163 (44.1)		245	14 (5.7)	97 (39.6)	134 (54.7)	
pT stage					**.016**					**.008**
T1‐2	105	19 (18.1)	26 (24.8)	60 (57.1)		51	0 (0.0)	12 (23.5)	39 (76.5)	
T3‐4	289	90 (31.1)	90 (31.1)	109 (37.7)		205	14 (6.8)	89 (43.4)	102 (49.8)	
pN stage					.149					.744
N0	166	52 (31.3)	38 (22.9)	76 (45.8)		121	4 (3.3)	49 (40.5)	68 (56.2)	
N1/2/3	228	57 (25.0)	78 (34.2)	93 (40.8)		135	10 (7.4)	52 (38.5)	73 (54.1)	
Adjuvant therapy					.290					.914
No	327	96 (29.4)	96 (29.4)	135 (41.3)		11	8 (5.8)	53 (38.4)	77 (55.8)	
Yes	67	13 (19.4)	20 (29.9)	34 (50.7)		245	6 (5.1)	48 (40.7)	64 (54.2)	

^a^ Median age.

^b^ Chi‐square test with Benjamini–Hochberg correction.

Abbreviations: ESCC, oesophageal squamous cell carcinoma; Centre A, Sun Yat‐sen University Cancer Center; Centre B, Cancer Hospital of Shantou University Medical College; TLS, tertiary lymphoid structure; LNs, lymph nodes.

### 2.2 Prognostic value of TLS maturation status in ESCCs from centre A

**FIGURE 1:**
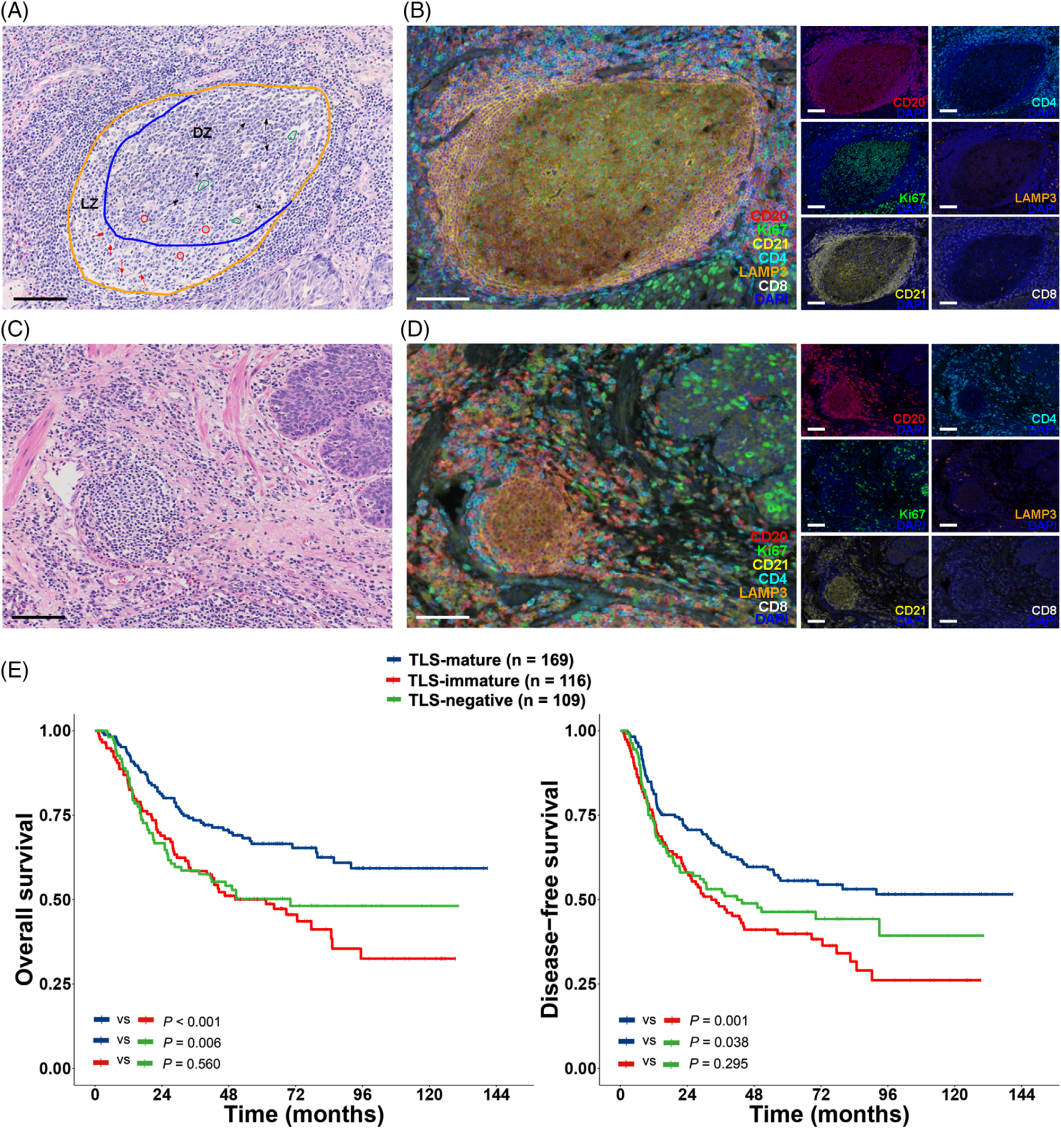
The maturation status of TLSs correlated with ESCC patient survival in the Centre A cohort. (A) Representative H&E‐stained image of an immature TLS. Scale bar, 100 μm. (B) mfIHC‐stained images of the immature TLS as in (A) with CD20, Ki67, CD21, CD4, LAMP3, CD8 and DAPI. Scale bar, 100 μm. (C) Representative H&E‐stained image of a mature TLS. The germinal centre of the mature TLS was circled with the orange line, which was separated into the dark zone (DZ) and the light zone (LZ) as shown by the blue line. Red circles, FDCs; red arrows, centrocytes; black arrows, centroblasts; green polygons, phagocytosis. Scale bar, 100 μm. (D) mfIHC‐stained images of the mature TLS as in (C) with CD20, Ki67, CD21, CD4, LAMP3, CD8 and DAPI. Scale bar, 100 μm. (E) Kaplan–Meier curves for overall and disease‐free survival of ESCC patients with TLS‐mature (*n* = 169), TLS‐immature (*n* = 116) and TLS‐negative (*n* = 109) tumours in the Centre A cohort. *p‐*Values by Kaplan–Meier analysis with a log‐rank test

A total of 1584 original H&E‐stained slides were obtained for the 394 ESCCs (average ± standard deviation: 4.020 ± 0.462 slides for each patient) in the Centre A cohort. Figure 1A shows an H&E‐stained image of a mature TLS, a secondary lymphoid follicle with germinal centre (GC) formation. GCs are highly dynamic structures with a network of follicular dendritic cells (FDCs) fully filled with centroblasts and centrocytes. They could be separated into two zones, the dark zone (DZ) dominated by centroblasts and the light zone (LZ) containing centrocytes and FDCs.^12^ Phagocytosis could be observed in both the DZ and LZ compartments. In contrast, immature TLSs were clusters of lymphocytes without GCs, as shown in Figure 1C. Follicular CD 20+ B cell zones and surrounding T (primarily CD4+ T) cell zones were observed in both mature and immature TLSs on multiplex fluorescent immunohistochemistry (mfIHC) images (Figure 1B,D). Staining for CD21 showed that dendritic protrusions of the FDCs were interlaced and formed a fingerprint‐like meshwork in the B cell zones of both mature and immature TLSs. LAMP3+ dendritic cells (DCs) were mainly located in the T cell zones of TLSs. Unlike immature TLSs, Ki67 was highly expressed in the GC of mature TLSs (Figure 1B,D).

After careful pathological evaluation, 106 cases (26.9%) in the Centre A cohort were TLS‐negative tumours. Among the 288 (73.1%) TLS‐positive cases, 169 (42.9%) had mature TLSs (defined as TLS‐mature tumours) and 119 (30.2%) had only immature TLSs (defined as TLS‐immature tumours).

TLS‐mature ESCCs exhibited significantly better survival than both TLS‐immature and TLS‐negative ESCCs (*p* < .05). However, no significant difference in survival between TLS‐immature and TLS‐negative tumours was observed (*p* > .05, Figure 1E). The 5‐year overall survival (OS) rates were 66.5%, 50.0% and 50.2% (*p* = .001) and the disease‐free survival (DFS) rates were 55.6%, 39.9% and 46.3% (*p* = .003) for patients with TLS‐mature, TLS‐immature and TLS‐negative ESCCs, respectively (Figure 1E). The presence of mature TLSs in primary ESCC tumours was associated with an earlier pT stage (*p* = .016) but no other clinicopathological factors (Table 1).

To clarify whether the spatial distribution of mature TLSs affects ESCC survivals, the existence and maturation status of TLSs were evaluated in the intratumoural and peritumoural regions of the 169 TLS‐mature ESCCs (Figure S1A). The location of mature TLSs did not affect ESCC OS and DFS (*p* > .05, Figure S1B).

Univariate Cox analysis identified that the presence of mature TLSs, female sex, pT1‐2 and pN0 were associated with improved DFS (*p* < .05) and the presence of mature TLSs, pT1‐2 and pN0 with improved OS (*p* < .05). Multivariate Cox analysis identified the presence of mature TLSs, pT and pN stages as independent prognostic factors for DFS (*p* < .05). For OS, the presence of mature TLSs and pN stage were significantly independent prognostic factors (*p* < .05, Table 2).

**TABLE 2:** Univariate and multivariate analyses for DFS and OS in ESCC patients in the Centre A and Centre B cohorts

	**DFS**				**OS**			
	**Centre A cohort**		**Centre B cohort**		**Centre A cohort**		**Centre B cohort**	
**Variable**	*p* Value	HR (95% CI)	*p* Value	HR (95% CI)	*p* Value	HR (95% CI)	*p* Value	HR (95% CI)
**Univariate analysis** ^a^								
Mature TLS (presence vs. absence)	**.001**	0.63 (0.47–0.83)	**.006**	0.59 (0.40–0.85)	**<.001**	0.55 (0.40–0.76)	**.025**	0.62 (0.41–0.94)
Age (>60 vs. ≤60)	.971	1.00 (0.76–1.31)	.069	1.44 (0.97–2.12)	.301	1.17 (0.87–1.59)	**.009**	1.81 (1.16–2.83)
Gender (female vs. male)	**.038**	0.68 (0.47–0.98)	.190	0.73 (0.45–1.17)	.241	0.79 (0.54–1.17)	.072	0.60 (0.34–1.05)
Tumour location								
Upper	‐	‐	‐	‐	‐	‐	‐	‐
Middle	.283	0.81 (0.54–1.20)	.919	1.03 (0.61–1.73)	.364	0.82 (0.54–1.26)	.527	1.21 (0.66–2.23)
Lower	.799	0.95 (0.62–1.45)	.396	1.30 (0.71–2.40)	.564	0.87 (0.54–1.40)	.107	1.76 (0.89–3.49)
Tumour differentiation								
Well	‐	‐			‐	‐	‐	‐
Moderate	.207	1.40 (0.83–2.35)	.242	1.29 (0.84–1.98)	.140	1.57 (0.86–2.85)	.471	1.19 (0.74–1.91)
Poor	.086	1.62 (0.93–2.82)	.309	1.31 (0.78–2.21)	.080	1.76 (0.93–3.30)	.347	1.32 (0.74–2.33)
pT stage (T3‐4 vs. T1‐2)	**.003**	1.69 (1.2–2.37)	.060	1.69 (0.98–2.91)	**.015**	1.58 (1.10–2.29)	**.017**	2.22 (1.15–4.30)
pN stage (N1‐3 vs. N0)	**<.001**	2.67 (1.95–3.64)	**<.001**	2.32 (1.55–3.48)	**<.001**	2.55 (1.81–3.59)	**<.001**	2.75 (1.73–4.37)
Number of retrieved LNs (≥15 vs. <15)	.357	0.77 (0.44–1.35)	.719	1.20 (0.44–3.26)	.136	0.65 (0.37–1.14)	.667	1.29 (0.41–4.07)
Adjuvant therapy (no vs. yes)	.334	0.84 (0.59–1.20)	**.003**	0.56 (0.38–0.81)	.543	1.14 (0.75–1.73)	**.007**	0.56 (0.36–0.85)
**Multivariate analysis^b^**								
Mature TLS (presence vs. absence)	**.008**	0.67 (0.50–0.90)	**.004**	0.57 (0.39–0.83)	**<.001**	0.56 (0.41–0.78)	**.04**	0.64 (0.42–0.98)
Age (>60 vs. ≤60)	‐	‐	‐	‐	‐	‐	**.01**	1.76 (1.12–2.75)
Gender (female vs. male)	‐	‐	‐	‐	‐	‐	‐	‐
pT stage (T3‐4 vs. T1‐2)	**.031**	1.46 (1.03–2.06)	‐	‐	‐	‐	‐	‐
pN stage (N1‐3 vs. N0)	**<.001**	2.57 (1.88–3.50)	**<.001**	2.37 (1.58–3.55)	**<.001**	2.52(1.79–3.55)	**<.001**	2.67 (1.66–4.30)
Adjuvant therapy (no vs. yes)	‐	‐	‐	‐	‐	‐	‐	‐

^a^ Univariate Cox proportional hazards regression.

^b^ Multivariate Cox proportional hazards regression with backward stepwise.

DFS, disease‐free survival; OS, overall survival; ESCC, oesophageal squamous cell carcinoma; Centre A, Sun Yat‐sen University Cancer Center; Centre B, Cancer Hospital of Shantou University Medical College; TLS, tertiary lymphoid structure; HR, hazard ratio; CI, confidence interval; LNs, lymph nodes.

### 2.3 Establishment and performance of the DL TLS classifier model

**FIGURE 2:**
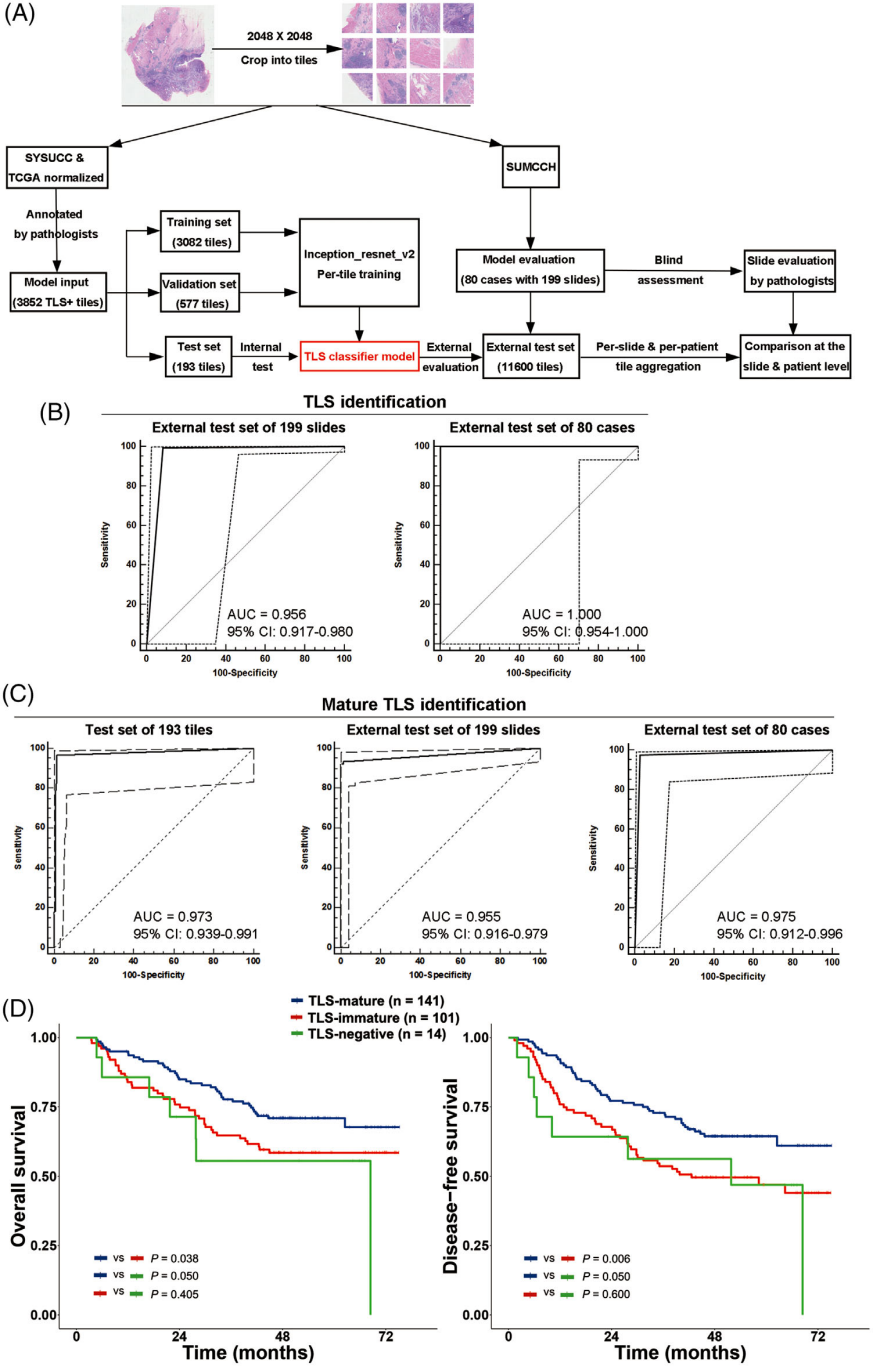
Establishment and evaluation of the deep‐learning (DL) TLS classifier model. (A) The data analysis workflow in detail. (B) ROC curves were used to evaluate the performance of the TLS classifier model in TLS identification at the slide‐ and patient‐level with 199 slides and 80 patients from the external test set. (C) ROC curves were used to evaluate the performance of the TLS classifier model in mature TLS identification at the tile‐, slide‐ and patient‐level with 193 tiles from the test set, 199 slides and 80 patients from the external test set. (D) Kaplan–Meier curves for overall and disease‐free survivals between ESCC patients with TLS‐mature (*n* = 141), TLS‐immature (*n* = 101) and TLS‐negative (*n* = 14) tumours evaluated by the DL TLS classifier model in the Centre B cohort. *p*‐Values by Kaplan–Meier analysis with a log‐rank test

A total of 3852 TLS‐positive tiles with mature or immature TLSs framed on from Centre A and The Cancer Genome Atlas (TCGA) H&E‐stained slides were used. With the 3082 tiles for training and 577 for validation processes, a DL TLS classifier using inception‐resnet‐v2 neural network^13^ was established to identify TLSs and evaluate their maturation status (Figure 2A). The classifier was tested on the test set of 193 tiles with an overall accuracy of 95.3% for TLS identification. The specificity and receiver operating characteristic (ROC) curve for TLS identification in the test set were not calculated because the 193 tiles were all TLS‐positive ones. The model performed well in TLS identification at the patient level, with an overall accuracy of 100% (Figure 2B; Table S1). An area under the curve (AUC) of 0.973 (95% confidence interval: 0.939–0.991) for mature TLS identification was achieved. Another set of 199 slides from 80 patients in the Centre B cohort, which included 11600 tiles, was used to externally test the TLS classifier model at the slide and patient levels. The values of AUC, accuracy and sensitivity for mature TLS identification at the slide level of the external test set decreased slightly. However, the model performed well at the patient level with high AUC, accuracy, precision, recall, specificity and F1 score^14^ for mature TLS detection (Figure 2C; Table S1).

### 2.4 Prognostic value of TLS maturation status evaluated by the DL TLS classifier in the centre B cohort

We applied our DL TLS classifier to identify TLSs and evaluate the maturation status of 256 ESCC patients based on 639 H&E‐stained slides (average ± standard deviation: 2.496 ± 0.501 slides for each patient) in the Centre B cohort and classified 141 (55.1%) ESCCs as TLS‐mature tumours, 101 (39.5%) as TLS‐immature tumours and 14 (5.5%) as TLS‐negative tumours. Similar to the results in the Centre A cohort, the absence of mature TLSs was associated with advanced tumour stage (pT3‐4) (*p* = .008, Table 1). The 5‐year OS rates were 70.9%, 58.5% and 55.6% (*p* = .043) and the 5‐year DFS rates were 64.4%, 47.0% and 46.9% (*p* = .011) for patients with TLS‐mature, TLS‐immature and TLS‐negative ESCCs, respectively (Figure 2D). TLS‐mature ESCCs exhibited significantly better survival than TLS‐immature (*p* = .038 and .006 for OS and DFS, respectively) and TLS‐negative ones (*p* = .050 for both OS and DFS), but there was no significant difference in survival between TLS‐immature and TLS‐negative tumours (*p* > .05 for both OS and DFS). The presence of mature TLSs was retained as an independent predictor for better DFS and OS (*p* < .05) in the Centre B cohort by multivariate Cox analysis (Table 2).

As mature and immature TLSs could co‐exist with varied numbers of TLS‐mature tumours, we obtained the exact numbers of mature and immature TLSs of the 141 TLS‐mature tumours in the Centre B cohort using the DL TLS classifier and calculated the densities of mature and immature TLSs and the proportion of mature to total TLSs (Figure S2A,B). Kaplan–Meier analyses with log‐rank test and univariate Cox regression analyses showed that neither the densities of mature or immature TLSs nor the proportion of mature to total TLS affected TLS‐mature ESCCs’ OS and DFS (*p* > .05) (Figure S2C–E; Table S2).

### 2.5 Spatial transcriptomic characteristics and immune repertoires of TLSs in ESCC

**FIGURE 3:**
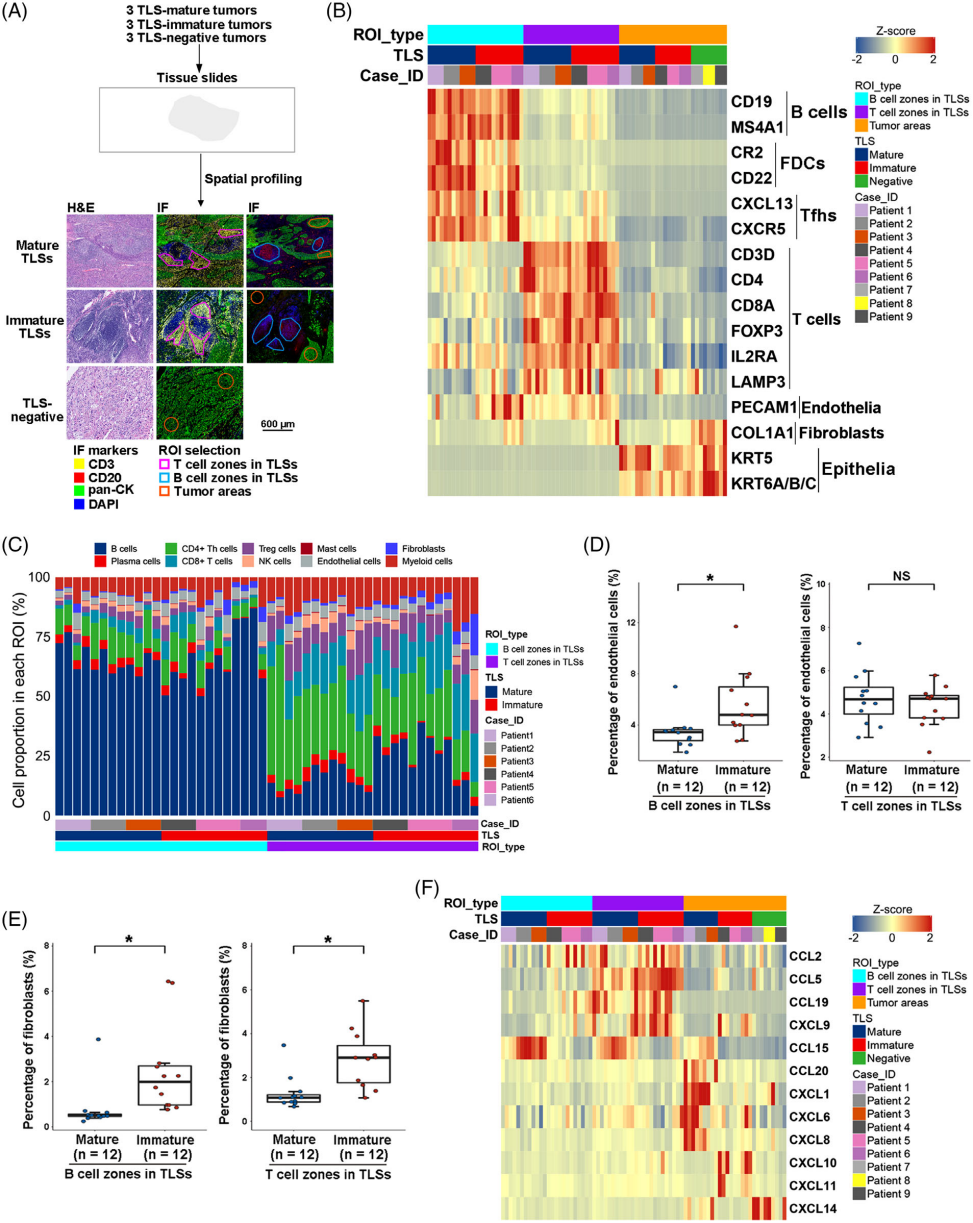
Spatial transcriptomic characteristics of TLSs and tumour areas in TLS‐mature, TLS‐immature and TLS‐absent ESCCs. (A) Workflow of the GeoMx DSP assay. (B) Heatmap showing the expression of marker genes for B cells (MS4A1 and CD19), FDCs (CR2 and CD22), Tfh cells (CXCL13 and CXCR5), T cells (CD3D, CD4 and CD8A), Treg cells (FOXP3 and IL2RA), mature DCs (LAMP3), endothelial cells (PECAM1), fibroblasts (COL1A1) and tumour cells (KRT5 and KRT6A/B/C) by DSP. (C) Distribution of cell proportion in each sequenced region of interest. (D) Box plots showing the differences of proportions of endothelial cells in B (left) and T (right) cell zones between mature (*n* = 12) and immature (*n* = 12) TLSs. (E) Box plots showing the differences of proportions of fibroblasts in B (left) and T (right) cell zones between mature (*n* = 12) and immature (*n* = 12) TLSs. (F) Heatmap showing differentially expressed chemokines in B and T cell zones between mature and immature TLSs and in tumour areas among TLS‐mature, TLS‐immature and TLS‐negative ESCCs

To further examine the cellular and molecular characteristics of TLSs at different maturation statuses and their potential effects on tumour cells, GeoMx DSP^15^ was used to perform highly multiplexed gene expression analysis with spatial resolution on 3 TLS‐mature, 3 TLS‐ immature and 3 TLS‐negative surgically resected ESCC primary tumours without pre‐operative chemotherapy or radiation (Figure 3A; Table S3). Transcriptome data for 1833 genes in 75 different spatially resolved regions of the region of interest (ROIs), including 24 for B cell zones in TLSs, 24 for T cell zones in TLSs and 27 for tumour areas, were obtained (Figure S3; Table S4). B cell zones of TLSs had high expression of B cell marker genes, such as CD19 and MS4A1 (Figure 3B; Table S5), and activation of B cell receptor‐related pathways (Figure S4A). On the contrary, T cell and subtype marker genes CD3D, CD4, CD8A and FOXP3 were more expressed (Figure 3B; Table S5) and T cell receptor‐related pathways were more activated (Figure S4B) in T cell zones of TLSs. Immune cell infiltration inferred by SpatialDecon^16^ demonstrated that B cells were the major cell type in the B cell zones and T cells [including CD4+ helper T (Th), regulatory T (Treg) and CD8+ T cells] were the major cell type in the T cell zones. However, both B and T cells could be detected in the T and B cell zones of TLSs (Figure 3C; Figure S4C and Table S6). The expression of other TLS markers, including CR2 and CD22 (markers for FDCs), CXCL13, CXCR5 (markers for follicular helper T [Tfh] cells) and LAMP3 (a marker for mature DCs), was also observed in the B or T cell zones of TLSs (Figure 3B). The expression of PECAM1 and COL1A1 (markers for endothelial cells and fibroblasts) and the existence of endothelial cells and fibroblasts inferred by SpatialDecon, was observed in the B and T cell zones of TLSs. The percentages of endothelial cells in B cell zones and fibroblasts in B and T cell zones of mature TLSs were higher in immature TLSs than in mature TLSs (Figure 3D,E).

The expression of chemokines, which were previously used to evaluate the existence of TLSs in cancers,^17^, ^18^ was evaluated in our DSP data (Table S7). The expression of CCL15 in B and T cell zones was significantly higher in mature TLSs and the expression of CCL2, CCL19 and CXCL9 in B cell zones and CCL5 in T cell zones was significantly higher in immature TLSs. The expression of CCL15, CCL20, CXCL1, CXCL6 and CXCL8 was higher in tumour areas from TLS‐mature ESCCs than in TLS‐immature and TLS‐negative ESCCs and the expression of CXCL9, CXCL10 and CXCL11 was higher in tumour areas from TLS‐immature ESCCs than in TLS‐mature and TLS‐negative ESCCs (fold change > 1.5, *p* < .05, Figure 3F; Table S8). The differentially expressed chemokines might be due to the varied proportions and activities of chemokine‐secreting cells in each sequenced ROI. Future analysis at the single‐cell level would aid in understanding the specific cell types involved in chemokine secretion.

**FIGURE 4:**
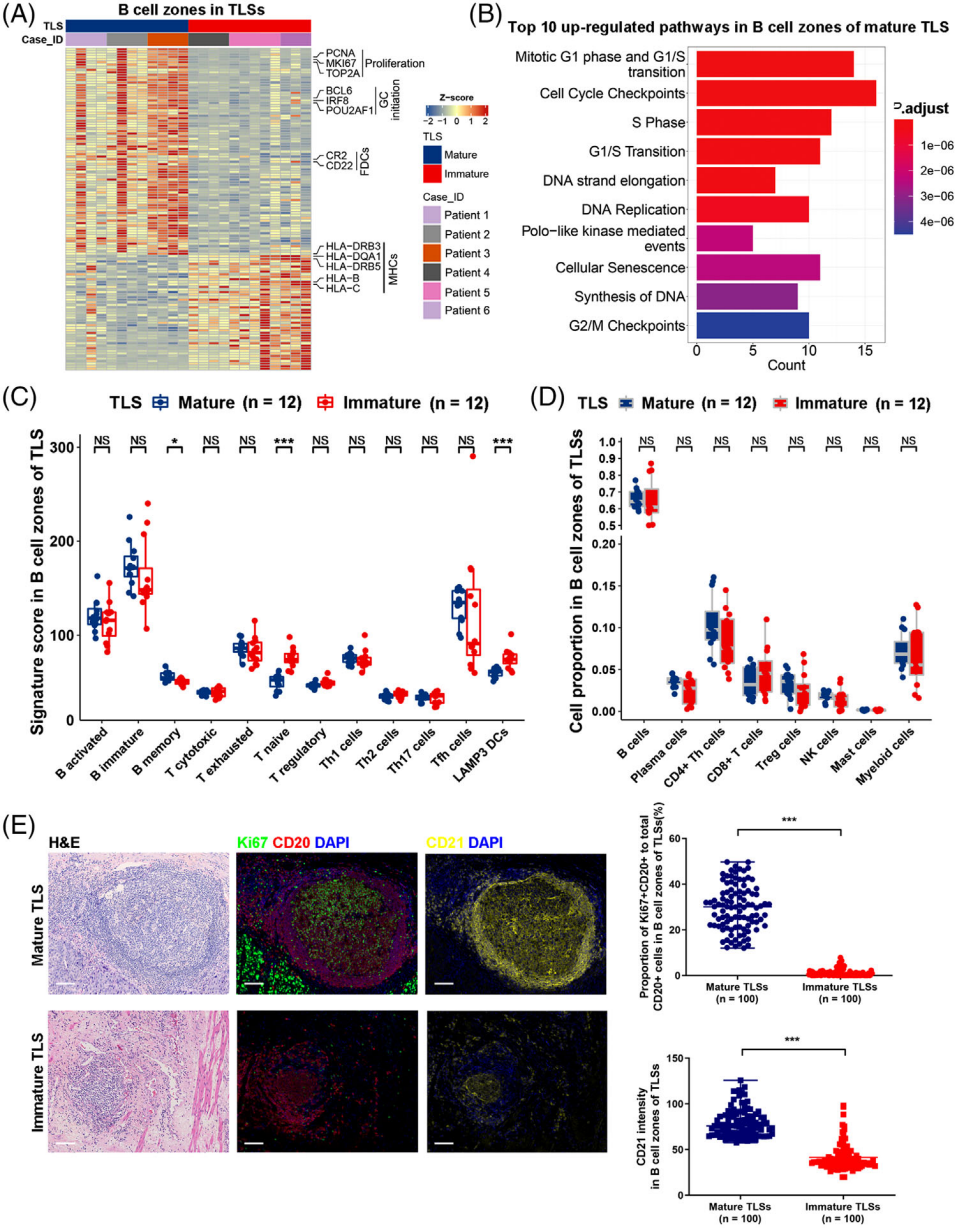
Transcriptomic characteristics of B cell zones in mature and immature TLSs. (A) Heatmap showing differentially expressed genes in B cell zones between mature and immature TLSs by DSP. (B) Histogram plot showing the top 10 upregulated pathways in B cell zones of mature TLSs compared to immature TLSs. (C) Box plots showing the differences in immune signature scores of B cell zones between mature (*n* = 12) and immature (*n* = 12) TLSs. (D) Box plots showing the differences in immune cell proportions in B cell zones between mature (*n* = 12) and immature (*n* = 12) TLSs. (E) Representative H&E‐stained images and mfIHC‐stained images with CD20, Ki67, CD21 and DAPI in mature and immature TLSs. Differences in the proportion of Ki67+CD20+ to total CD20+ cells and CD21 intensity were compared in the B cell zones between mature (*n* = 100) and immature (*n* = 100) TLSs. *, *p* < .05; ***, *p* < .001 by Mann–Whitney U test

For B cell zones in TLSs, mature TLSs had higher expression of proliferative markers PCNA, MKI6 and TOP2A (Figure 4A; Table S9) and higher activity of proliferation‐related pathways than immature TLSs (Figure 4B). The master regulators of GC initiation, BCL6, IRF8 and POU2AF1^19^ were expressed at higher levels in B cell zones from mature TLSs than from immature TLSs (Figure 4A; Table S9). The B cell zones of mature TLSs were characterised by an increased B memory signature (Figure 4C; Table S10) with higher expression of the affinity maturation gene AICDA^10^ (Table S9), suggesting long‐lived humoral immunity in ESCCs with mature TLSs.^19^ However, no significant differences in B and plasma cell proportions between B cell zones from mature and immature TLSs were observed (Figure 4D). Higher expression of the FDC markers CR2 and CD22 (Figure 4A; Table S9) were observed in the B cell zones of mature TLSs, which suggests important roles of FDCs in GC formation during TLS maturation.^20^, ^21^ A lower LAMP3+ DC signature was observed in B cell zones of mature TLSs than immature TLSs (Figure 4C), probably due to the decreased expression of LAMP3+ DC signature gene CCL19 in B cell zones of mature TLSs. However, there were few LAMP3+ DCs in the B cell zones of mature TLSs as shown by mfIHC (Figure 1B,D), and there was no significant difference in LAMP3 expression in B cell zones between mature and immature TLSs. Therefore, the decreased expression of CCL19 in the B cell zones of mature TLSs was probably due to the decreased percentages of other CCL19‐secreting cells, such as fibroblasts^22^ and endothelial cells.^23^ T cells in the B cell zones of immature TLSs were with higher expression of naïve T cell marker SELL (Table S9) and an increased T naïve signature (Figure 4C; Table S10). Antigens are presented by FDCs to B cells and by B cells and DCs to T cells in the B cell zones of TLSs.^20^ A broad decrease in the expression of major histocompatibility complex (MHC) I and II molecules (Table S7) in the B cell zones of mature TLSs was observed compared to immature TLSs (Figure 4A; Table S9). By quantification of the percentages of Ki67+CD20+ cells and CD21 intensity in the B cell zones of 100 mature and 100 immature TLSs from ESCCs by mfIHC, a higher ratio of Ki67+CD20+ cells to total CD20+ cells (*p* < .001) and a higher intensity of CD21 expression (*p* < .001) were observed in the B cell zones of mature TLSs than immature TLSs (Figure 4E).

**FIGURE 5:**
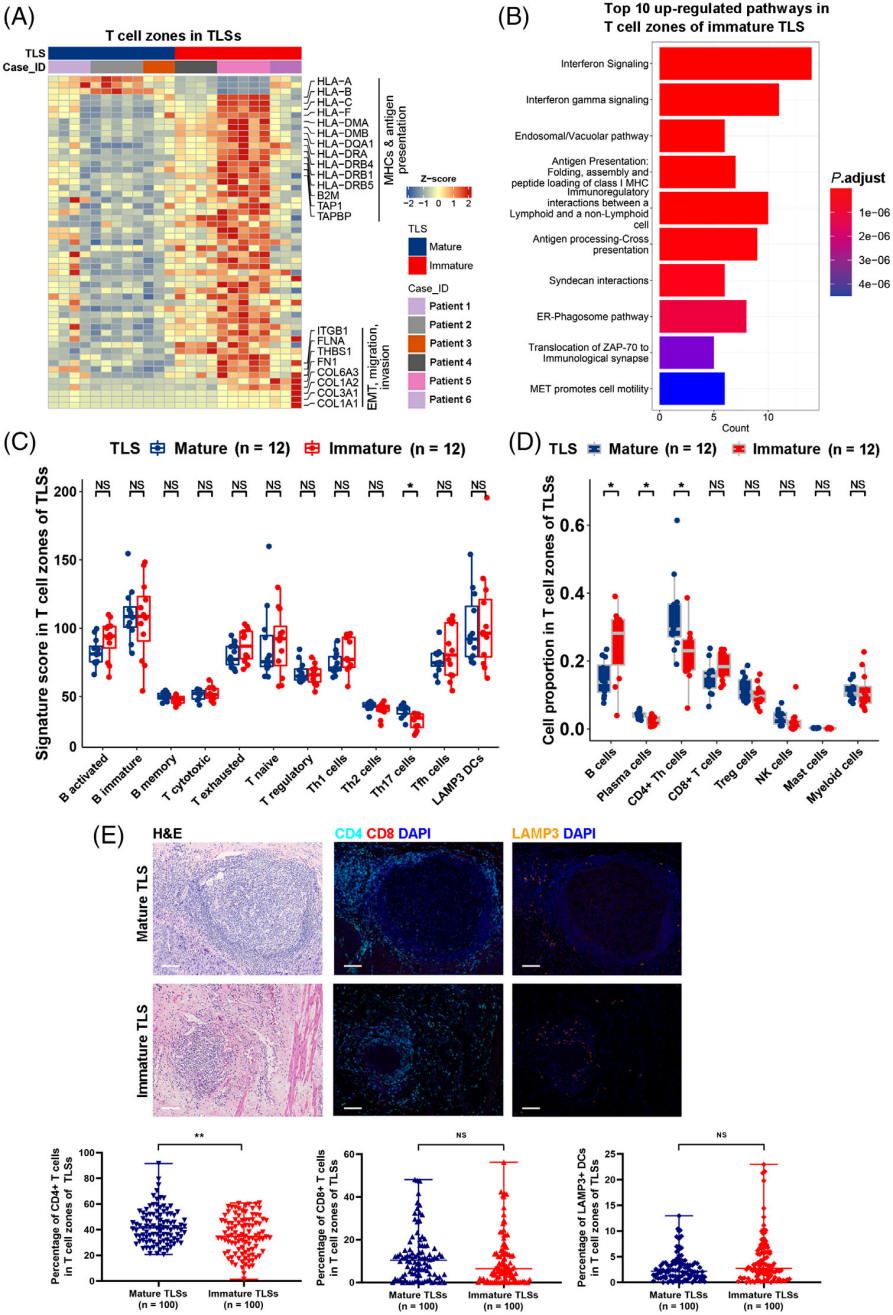
Transcriptomic characteristics of T cell zones in mature and immature TLSs. (A) Heatmap showing differentially expressed genes in T cell zones between mature and immature TLSs by DSP. (B) Histogram plot showing the top 10 upregulated pathways in T cell zones of immature TLSs compared to mature TLSs. (C) Box plots showing the differences in immune signature scores of T cell zones between mature (*n* = 12) and immature (*n* = 12) TLSs. (D) Box plots showing the differences in immune cell proportions in T cell zones between mature (*n* = 12) and immature (*n* = 12) TLSs. (E) Representative H&E‐stained images and mfIHC‐stained images with CD4, CD8, LAMP3 and DAPI of mature and immature TLSs. Differences in the percentages of CD4+ and CD8+ T cells were compared in the T cell zones between mature (*n* = 100) and immature (*n* = 100) TLSs. *, *p* < .05; **, *p* < .01; NS, not significant by Mann–Whitney U test

B cell clusters in TLSs were surrounded by T cells, primarily CD4+ Th cells (Figures 1B,D and 3C). Increased expression of MHC I and II molecules (Figure 5A; Table S11) and genes involved in antigen presentation such as B2M, TAP1 and TAPBP, as well as up‐regulated pathways of antigen presentation (Figure 5B) were observed in the T cell zones of immature TLSs as compared to mature TLSs. Expression of collagen genes COL1A1, COL1A2, COL3A1 and COL6A3 increased in immature TLSs (Figure 5A; Table S11), probably due to the increased proportion of fibroblasts in the B cell zones of immature TLSs (Figure 3E). A higher proportion of CD4+ Th cells with a higher Th17 signature calculated by the expression of Th17 signature genes, such as IL17s and IL22 (Table S10), was observed in the T cell zones of mature TLSs compared to immature TLSs (Figure 5C,D). No significant difference in the proportion of CD8+ T cells in T cell zones was observed between mature and immature TLSs. B and plasma cells existed in the T cell zones, with a higher proportion of plasma cells and a lower proportion of B cells in mature TLSs than in immature TLSs (Figure 5D). The LAMP3+ DC signature (Table S10) in T cell zones was not significantly different between mature and immature TLSs (Figure 5C). By quantification of the percentages of CD4+ and CD8+ T cells and LAMP3+ DCs in the T cell zones of 100 mature and 100 immature TLSs from ESCCs by mfIHC, higher percentages of CD4+ T cells (*p* = .004), but not CD8+ T cells (*p* = .168) and LAMP3+ DCs (*p* = .092), were observed in the T cell zones of mature TLSs compared to immature TLSs (Figure 5E).

### 2.6 Mature TLSs shaped the ESCC intratumoural immune microenvironment

**FIGURE 6:**
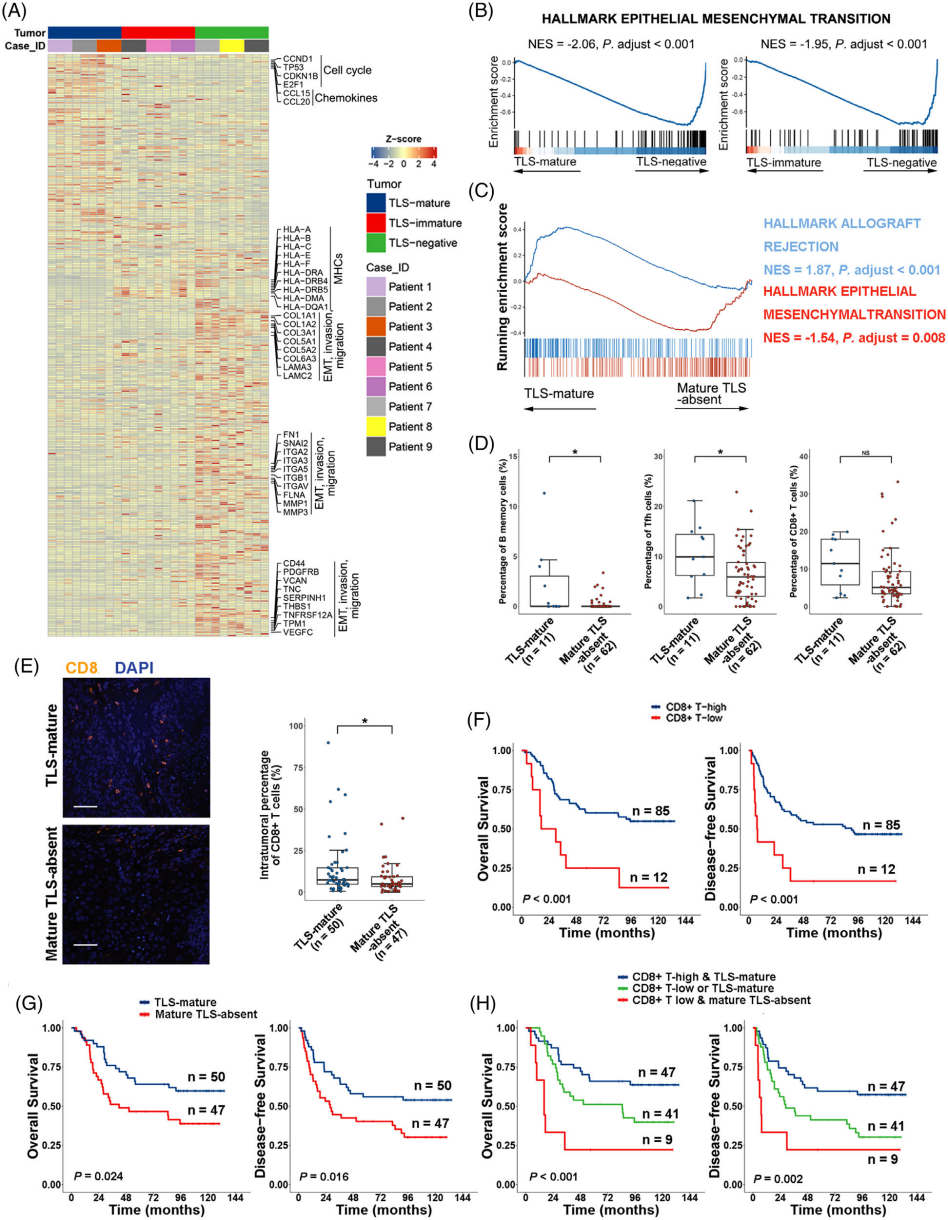
Characteristics of tumour areas and the intratumoural immune microenvironment of TLS‐mature, TLS‐immature and TLS‐negative ESCCs. (A) Heatmap showing differentially expressed genes among tumour areas of TLS‐mature, TLS‐immature and TLS‐negative ESCCs by DSP. (B) Enrichment plots showing high enrichment of the EMT pathway in tumour areas of TLS‐negative ESCCs as compared to TLS‐mature and TLS‐immature ESCCs, respectively. (C) Enrichment plots of the allograft rejection and EMT pathways between TLS‐mature and mature TLS‐absent TCGA ESCC samples. (D) Box plots showing the percentages of B memory, Tfh and CD8+ T cells estimated by CIBERSORT in TCGA TLS‐mature (*n* = 11) and mature TLS‐absent (*n* = 62) ESCC samples. *, *p* < .05; NS, not significant by Mann–Whitney U test. (E) Representative mfIHC‐stained images of TLS‐mature and mature TLS‐absent ESCCs with CD8 and DAPI. Scale bar, 50 μm. Differences in the percentages of intratumoural CD8+ T cells were compared between TLS‐mature (*n* = 50) and mature TLS‐absent (*n* = 47) ESCCs. *, *p* < .05 by Mann–Whitney U test. (F,G) Kaplan–Meier curves for overall and disease‐free survivals of ESCC patients with high (*n* = 85) and low (*n* = 12) intratumoural CD8+ T infiltration (F) and TLS‐mature (*n* = 50) and mature TLS‐absent (*n* = 47) tumours (G). *p* Values by Kaplan–Meier analysis with a log‐rank test. (H) Kaplan–Meier curves for overall and disease‐free survival of ESCCs stratified by combining intratumoural CD8+ T infiltration and TLS maturation status. *n* = 47 for high CD8+ T infiltration and TLS‐mature tumours; *n* = 41 for low CD8+ T infiltration or TLS‐mature tumours; *n* = 9 for low CD8+ T infiltration and mature TLS‐absent tumours. *p* Values by Kaplan–Meier analysis with a log‐rank test

We next evaluated the gene expression of pan‐CK+ tumour areas from TLS‐mature, TLS‐immature and TLS‐negative ESCCs (Figure 6A) in the GeoMx DSP data. The expression of a series of G1/S cell cycle‐related genes (CCND1, TP53, CDKN1B and E2F1) and chemokines (CCL15 and CCL20) was higher in tumour areas of TLS‐mature tumours than in TLS‐immature and TLS‐negative tumours (Figure 6A; Table S12), which underlies the characteristics of TLS‐mature tumour cells in the cell cycle and immune regulation. The expression of MHC I and II molecules (Table S7) was higher in tumour areas of TLS‐immature tumours than TLS‐negative tumours, both of which were higher than TLS‐mature tumours (Figure 6A; Table S12). A series of genes involved in epithelial‐mesenchymal transition (EMT) and tumour invasion and migration (Table S7) were expressed at higher levels in tumour areas of TLS‐negative ESCCs than TLS‐mature and TLS‐immature ESCCs, such as the mesenchymal markers FN1 (Fibronectin 1), ACTA2 and SNAI2, basement membrane components collagens, laminins, integrins and filamins,^24^ MMPs and so on (Figure 6A; Table S12). The EMT pathway was shown to be highly enriched in tumor areas of TLS‐negative ESCCs compared to either TLS‐immature or TLS‐mature ESCCs by gene set enrichment analysis (GSEA)^25^ (Figure 6B).

Bulk RNA‐sequencing and clinicopathological data of 73 TCGA ESCC patients who met our recruiting criteria were analysed, and TLS presence and maturation status were evaluated pathologically on H&E‐stained slides (Table S13). Using GSEA, the EMT pathway was highly enriched in mature TLS‐absent ESCCs (Figure 6C). However, the pathway of allograft rejection was enriched in TLS‐mature ESCCs (Figure 6C), which was probably related to the higher reactivity of immune cells to cancer cell‐encoded neoepitopes in TLS‐mature ESCCs. Higher percentages of B memory and Tfh cells were identified in TCGA TLS‐mature ESCC tissues than in mature TLS‐absent tissues, as estimated by CIBERSORT (Figure 6D; Figure S5A).^26^ A higher percentage of CD8+ T cells was also observed in TLS‐mature ESCCs, although statistical significance was not achieved, which suggests a possible correlation between TLSs and the ESCC intratumoural immune microenvironment. No significant difference in survival between TLS‐mature and mature TLS‐absent ESCC patients was observed in the TCGA cohort (Figure S5B). Pathological examination found that most TCGA ESCC specimens were superficially sampled with only one H&E‐stained slide available, which introduced the possibility of accidental omission errors in TLS evaluation.

To further examine the intratumoural adaptive immune response that might be affected by the presence and maturation of TLSs in ESCCs, we used data from a previous study on immune infiltration in ESCCs from Centre A by mfIHC (Figure S6A,B).^7^ Among the 279 ESCCs previously studied, 97 had TLS presence and maturation evaluation in the current study. Fifty (51.5%) of these ESCCs were TLS‐mature ESCCs and 47 (48.5%) of these ESCCs were mature TLS‐absent ESCCs. The tumour regions used for mfIHC were tumour cores without TLS presence. The intratumoural CD8+ T cell infiltration from TLS‐mature ESCCs was higher than that from mature TLS‐absent ESCCs (*p =* .017, Figure 6E). However, CD4+ Th, Treg and memory T cells, natural killer cells, DCs and macrophages exhibited no difference in infiltration between TLS‐mature and mature TLS‐absent ESCCs (Figure S6C). For the 97 ESCC patients, survival analyses revealed that high infiltration of CD8+ T cells or the presence of mature TLSs was associated with improved patient outcomes (Figure 6F,G). The combination of mature TLS presence and high CD8+ T cell infiltration was associated with the best prognosis, and the absence of mature TLSs and low CD8+ T cell infiltration with the worst prognosis (Figure 6H).

## 3 DISCUSSION

TLSs are aggregates of immune (mostly B and T) cells arising in response to immunological stimuli. There are differing reports of anti‐ and pro‐tumoural roles of TLSs in different types of cancers, which reflects the multiple roles of TLSs in tumour development and progression.^10^ Tumour TLSs vary widely in maturation state. The existence of TLSs correlated with patient survival in endometrial cancers,^27^ head and neck squamous cell carcinomas,^28^ metastatic melanomas^29^ and non‐functional pancreatic neuroendocrine tumours,^30^ regardless of their maturation status. However, Masuda et al.^31^ recently showed that the maturity, spatial distribution and prognostic impact of TLSs were significantly different between the two types of genitourinary cancers, clear‐cell renal cell cancer and bladder cancer, which suggests that the effects of TLSs on tumours were tumour type specific. We found that only the presence of mature TLSs predicted better survival in ESCCs, which is similar to pancreatic cancers,^32^ lung squamous cell carcinomas^33^ and colorectal cancers.^34^ The density of mature TLSs, as well as the ratio of mature to total TLSs, did not affect ESCC prognosis, which is different from previous reports in other types of cancers.^35^

DL‐based artificial intelligence has been developed to perform all kinds of work in tumour pathology.^36^, ^37^ Routine pathological examinations of TLSs are time‐consuming and include risks of perceptual bias and inter‐reader variability. Therefore, we established a DL TLS classifier model to resolve these problems by standardisation and training, which provided satisfactory results for mature TLS detection on H&E‐stained ESCC slides. Notably, our TLS classifier model performed prognostic stratification of ESCC in the external validation Centre B cohort, which proved to be a helpful tool for facilitating routine tumour TLS identification and maturation evaluation and stratifying prognosis in slide diagnosis. A well‐known disadvantage of the DL model is its black‐box nature, and the image features contributing to the prediction are hardly interpretable. The DL TLS classifier uses small‐scale features of histological images with undetermined biological correlates. Future studies using post hoc methods or handcrafted machine learning approaches would help increase the biological interpretability of the DL model.^38^

Evaluation of TLS presence and maturation on the H&E‐stained tissue sections is an effective approach. However, there might be several issues affecting its accuracy, such as the location and number of tumour samples and the planes of sectioning on tissue blocks. Therefore, we emphasised that all available H&E‐stained slides from multiple samples of the whole tumours used for routine pathological diagnosis should be adopted for precise TLS evaluation. Besides, the development of potent TLS detection and maturation evaluation assays based on bulk tumour tissues is still needed. The existence of TLSs was reported to be evaluated based on chemokine signature expression using bulk RNA‐sequencing data,^17^, ^18^ which would help avoid accidental inclination induced by tissue sectioning. In the present study, we found that TLS‐mature ESCCs could be differentiated from mature TLS‐absent ESCCs based on the expression of a series of chemokines, such as CCL15, CCL20, CXCL1 and CXCL8. Further studies to clarify the functions of specific cell types in chemokine secretion and their changes during TLS formation and maturation would aid in the application of chemokine expression signatures in TLS evaluation in ESCCs.

The prognostic value of mature TLSs in ESCCs may be primarily attributed to their cellular and molecular characteristics. Compared to immature TLSs, B cells within the GC of mature TLSs are highly proliferative, which is a prerequisite for generating mutant clones that have a broad range of affinities for immunizing antigens.^19^ As we found in the GeoMx DSP data, the B memory signature and proportion of plasma cells increased in mature TLSs, which would express a highly selected antibody repertoire and activate the complement pathway and trigger antibody‐dependent cellular cytotoxicity and cell killing to exert anti‐tumour effects.^39^ In addition to the changes of B cells, the T cell zones of mature TLSs exhibited a higher proportion of CD4+ Th cells with higher Th17 signatures than immature TLSs. Th17 cells and their cytokines have been reported to contribute to the development of TLSs in chronic inflammatory tissues,^40^, ^41^ such as damaged epithelia during the development of ESCC.^42^ The co‐existence of T cells with B cells and DCs in TLSs may provide a privilege for B cells and DCs to present tumour antigens to T cells, which would result in T cell activation.

Intratumoural CD8+ T cell infiltration was increased in TLS‐mature ESCCs, which is a significant factor correlating with prolonged ESCC survival.^7^ Whether TLS B cells or DCs cross‐present tumour antigens to CD8+ T cells or whether CD4+ Th cells are involved in the generation of CD8+ cytotoxic T cell responses inside or outside TLSs have not been settled.^10^ As decreased expression of MHC I antigen presentation molecules was observed in mature TLSs compared to immature TLSs, we speculated that increased CD4+ Th cells, which were activated with Th17 signatures in mature TLSs, may take part in priming and enhancing CD8+ T cells outside TLSs in ESCC tissues. Th17 cells positively correlated with CD8+ T infiltration and were associated with better survival in ESCCs.^43^ The association of the combination of TLS maturation status and CD8+ T cell infiltration with the best outcome indicated the importance of coordination between the cellular and humoral arms of the adaptive immune system in anti‐tumour immune response.^10^

Taken together, our study demonstrated that mature TLSs in ESCC facilitated immune cell cooperation and provided a privileged micro‐niche for B cells undergoing full differentiation, which further induced CD4+ T cell activation in TLSs and effective recruitment of CD8+ T cells in tumour regions to generate and sustain effective and memory anti‐tumour humoral and cellular immunity, and improve ESCC prognosis. Further studies to elucidate the detailed interactions between subsets or different immune cells in TLSs and ESCC TME are warranted. The present study is a springboard for future understanding of the biology of B cells and TLSs in ESCC and provides support for the exploration of new therapeutic opportunities.

## 4 METHODS AND MATERIALS

### 4.1 Patient selection

Pathologically confirmed thoracic ESCC patients undergoing complete surgical resection were recruited from Centre A between 2008 and 2017 and from Centre B between 2015 and 2017. These patients received complete tumour resection via McKeown, Ivor Lewis or minimally invasive oesophagectomy and two‐field lymphadenectomy with no preoperative chemo‐ or radiotherapy. All cases were pathologically staged according to the Eighth Edition American Joint Committee on Cancer tumour‐node‐metastasis staging system.

ESCC patients from TCGA who met the above criteria were also recruited, and updated clinical data were downloaded from the Genomic Data Commons (https://portal.gdc.cancer.gov/) using the R package TCGAbiolinks.^44^

OS was defined as the time from surgery to death or last follow‐up. DFS was defined as the time from surgery to the first recurrence or last follow‐up.

### 4.2 Pathological examination of TLSs

All of the available H&E‐stained slides of primary ESCC tumours in the Centre A cohort used for routine pathological diagnosis and the scanned whole slide images of H&E‐stained slides of primary ESCC tumours in the TCGA cohort (downloaded from https://portal.gdc.cancer.gov/) were used for TLS evaluation independently by three pathologists who were blind to the pathological diagnoses and disease outcomes. TLSs were evaluated on the whole H&E‐stained slides, and were classified based on their maturation status, as described previously^10^: (i) mature TLSs: secondary lymphoid follicles with GC formation. GCs are highly dynamic structures with a network of FDCs fully filled with centroblasts and centrocytes. GCs could be separated into two zones, the DZ dominated by centroblasts and the LZ containing centrocytes and FDCs. Extensive apoptosis and phagocytosis phenomena could be observed in both the DZ and LZ compartments; (ii) immature TLSs, loose, ill‐defined clusters of lymphoid aggregates or oval‐shaped clusters of lymphocytes without GC. The ESCC primary tumours were stratified according to the existence and maturation status of TLSs on each patient's H&E‐stained slides: TLS‐mature tumours with at least one mature TLS in any slide of the tumours, TLS‐immature tumours with at least one immature TLS but no mature TLS in any slide of the tumours and TLS‐negative tumours with neither mature nor immature TLS in all slides of the tumours. The TLS‐immature and TLS‐negative cases were called mature TLS‐absent tumours.

### 4.3 Establishment of a DL TLS classifier model

H&E‐stained slides of the Centre A and Centre B cohorts were scanned with the Vectra Polaris Automated Quantitative Pathology Imaging System (Perkin Elmer, Waltham, MA) at 40× magnification. Whole slide images from the Centre A, Centre B and TCGA cohorts were cropped into non‐overlapping tiles (2048 μm × 2048 μm) using QuPath (v 0.2.3).^45^ TCGA tiles were colour normalised as described previously.^46^ The tiles from the Centre A cohort and TCGA dataset were thoroughly annotated by pathologists, and each mature or immature TLS was framed on the tiles using LabelImg (v1.5.1). All tiles containing at least one mature or immature TLS were randomly split into training, validation and testing sets at ratios of 0.8, 0.15 and 0.05 and used for TLS classifier establishment.

The convolutional neural network inception‐resnet‐v2^13^ was used to establish the TLS classifier, which intended to identify TLSs and further discriminate between mature and immature TLSs on H&E‐stained slides’ images. The network was initialised using default weights transferred from the Macrosoft COCO dataset (http://mscoco.org/), which was then fine‐tuned with our dataset. The initial learning rate was 0.0003, and the optimizer was Momentum with a value of 0.9.^47^ The training process lasted for 200 000 steps. A set composed of all tiles from 199 H&E‐stained slides of 80 ESCC patients randomly selected from the Centre B cohort was used as an external test for the TLS classifier. All learning and testing were implemented using the TensorFlow library (v1.10.0, https://github.com/tensorflow/models) on a PowerEdge T630 (Dell, Round Rock, TX) with two GTX 1080 Ti graphics cards (NVIDIA, Santa Clara, CA).

The outputs of the TLS classifier model were tile‐level predictions. When any tile cropped from one slide was assessed as a TLS‐mature tile, the slide was regarded as a TLS‐mature slide. A slide was regarded as a TLS‐immature slide when at least one immature TLS but no mature TLS was identified in any tile cropped from the slide. A TLS‐negative slide lacked both mature and immature TLS identification. Slide‐level diagnoses were then aggregated into patient‐level diagnoses in a similar fashion. Ultimately, we collected the TLS evaluation results of the external test set from pathologists and compared them with the results from the TLS classifier model at the slide level and patient level for model performance evaluation. The ROC curve and the AUC, the accuracy, precision, recall, specificity and F1 score,^14^ were calculated to evaluate the value of the TLS classifier model in TLS identification and maturation evaluation.

### 4.4 GeoMX DSP

After conventional deparaffinization and rehydration, 4‐μm formalin‐fixed paraffin‐embedded (FFPE) tissue slides were hybridised with probes in the Cancer Transcriptome Atlas panel (NanoString, Seattle, WA) at 37°C overnight and incubated with fluorescent antibodies for pan‐CK (NanoString), CD3 (Roche, Indianapolis, IN) and CD20 (ABclonal, Wuhan, China). Spatially resolved ROIs, including T cell zones (the CD3+ cell‐clustered regions in the outer layer of TLSs) and B cell zones (the CD20+ and CD3‐ cell‐clustered regions in the centre of TLSs) in TLSs, and pan‐CK+ tumour areas outside TLSs, were selected on the slides based on the fluorescent markers and consecutive H&E‐stained slides. Conjugated target‐specific oligos of each ROI were collected in 96‐well plates. Library preparation was performed according to the manufacturer's instructions (NanoString) and sequenced on a NextSeq 550 (Illumina, San Diego, CA).

After quality check, raw counts were normalized with the Q3 normalization method, which was further used for quantifying cell populations in each ROI using SpatialDecon with the reference safeTME matrix.^16^ Immune signatures were calculated based on normalized data and were used to evaluate the activity and characteristics of immune cells (Table S10).^48^, ^49^, ^50^, ^51^ The raw count data was normalised and analysed by DESeq2 (v.1.28.1)^52^ to identify differentially expressed genes with *P* values adjusted with Benjamini–Hochberg correction for multiple tests (fold change ≥1.5 or ≤−1.5 and an adjusted *P* value <.05). For heatmap, a Z‐score normalisation is performed on the normalised expression data across samples for each gene. Pathway enrichment of differentially expressed genes in specific ROIs was analysed using the ReactomePA R package (v1.30.0)^53^ with *P* values adjusted with Benjamini–Hochberg correction for multiple tests.

### 4.5 TCGA data analysis

The clinical and RNA sequencing data of ESCC patients in TCGA were downloaded from https://portal.gdc.cancer.gov/. GSEA was performed on the 50 hallmark pathways in the Molecular Signature Database (v7.1).^25^ Immune cell infiltration was estimated with transcriptomic data using CIBERSORT.^26^

### 4.6 mfIHC staining

The compositions of major immune cells in TLSs of ESCCs were examined on FFPE ESCC slides using mfIHC and the PANO Multiplex IHC kit (Panovue, BJ, China) with antibodies for CD20 (Abcam, Boston, MA), CD21 (Abcam), Ki67 (Abcam), CD4 (Abcam), CD8 (ZSGB‐BIO, Beijing, China), LAMP3 (Abcam) and 4ʹ‐6ʹ‐diamidino‐2‐phenylindole (DAPI) as described previously.^7^ The stained slides were scanned using a Vectra Polaris Automated Quantitative Pathology Imaging System and batch analysed with HALO imaging analysis software (Indica Labs, Corrales, NM). Each TLS was segmented into a B cell zone (CD20+ cell and CD21+ cell‐clustered region) and a T cell zone (CD4+ cell and CD8+ cell‐clustered region). Cells in TLSs were phenotyped as B cells (CD20+), FDCs (CD21+), proliferating cells (Ki67+), CD4+ T cells (CD4+), CD8+T cells (CD8+) and mature DCs (LAMP3+). The intensity for each marker was recorded. The intratumoural infiltration of CD8+ T cells, CD4+ Th cells, Treg cells, memory T cells, natural killer cells, DCs and macrophages were evaluated on ESCC tissue microarrays as previously described.^7^

### 4.7 Statistical analysis

All statistical analyses were performed using R (v4.0.1). The correlation between TLS status and clinicopathological parameters was analysed with a Chi‐square test. Survival curves were calculated with Kaplan–Meier analysis and the log‐rank test. Independent prognostic factors were identified using univariate and multivariate Cox proportional hazards regression analyses with a backward stepwise procedure. The immune cell population and immune signature between two or three groups were compared with the Mann–Whitney U test and the Kruskal–Wallis test. All *P* values were adjusted with the Benjamini–Hochberg correction for multiple tests. Adjusted two‐sided *P* values less than .05 were considered statistically significant.

## CONFLICT OF INTEREST

The authors declare no conflict of interest.

## Supporting information

Figures information

Tables 1‐3 information

Table S4 information

Table S5 information

Table S6 information

Table S7 information

Table S8 information

Table S9 information

Table S10 information

Table S11 information

Table S12 information

Table S13 information
