# Supplementary material for: The prognostic value and molecular properties of tertiary lymphoid structures in oesophageal squamous cell carcinoma
Source: Clin Transl Med. 2022 Oct 17;12(10):e1074. doi: 10.1002/ctm2.1074 (PMC9574489; doi:10.1002/ctm2.1074)
Supplement: Supplementary file 1 — Figures information [file CTM2-12-e1074-s005.docx]

**Supplementary figures**

**
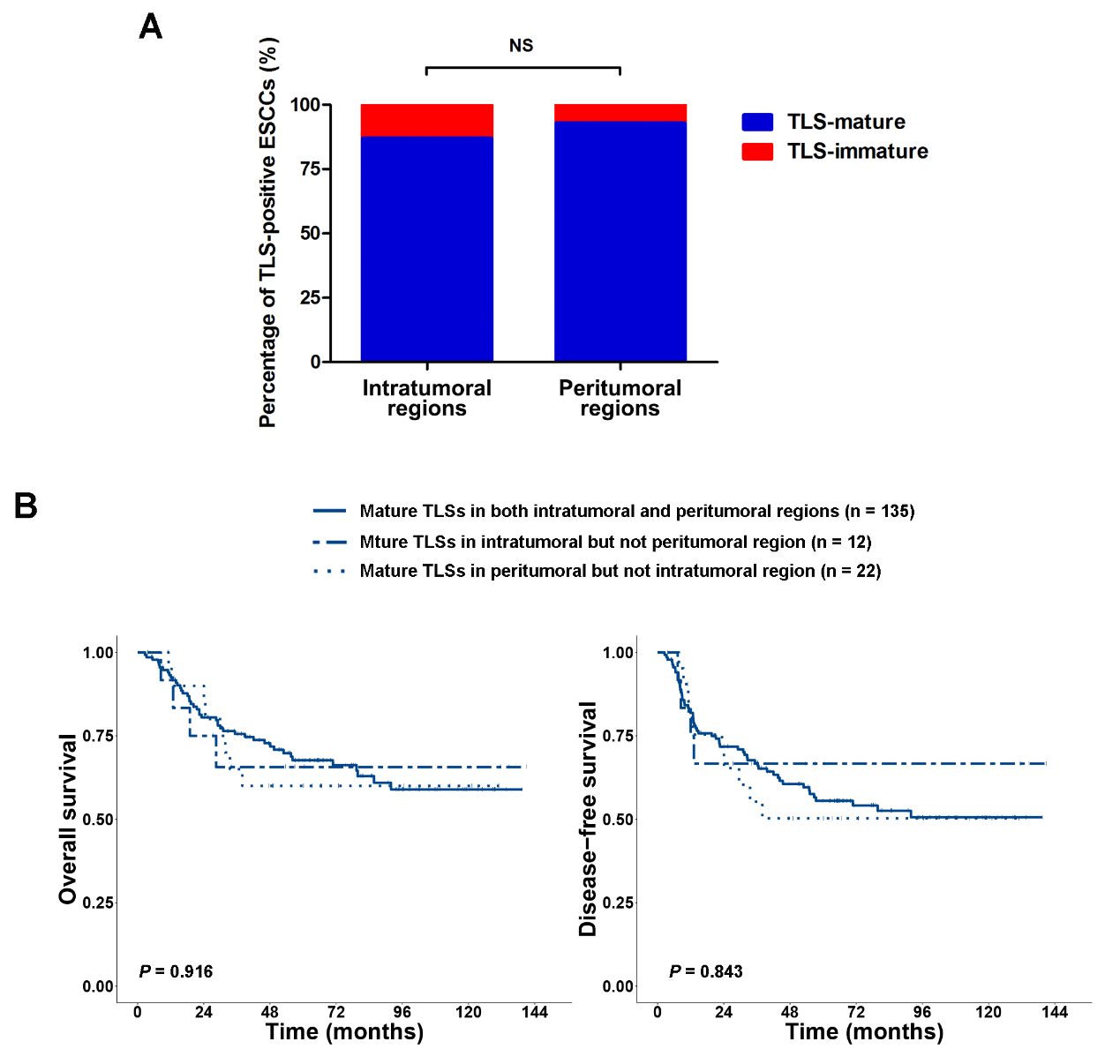
**

**Figure S1 Distribution and impact on survival of intratumoral and peritumoral mature TLSs in ESCC patients.** (A) Barplot showing the distribution of mature and immature TLSs in the intratumoral and peritumoral regions of 169 TLS-mature ESCCs in the Center A cohort. NS, not significant by McNemer test. (B) Kaplan-Meier curves for overall and disease-free survivals among 169 TLS-mature ESCCs in the Center A cohort with varied mature TLS locations. *P* values by Kaplan-Meier analysis with log-rank test.

**
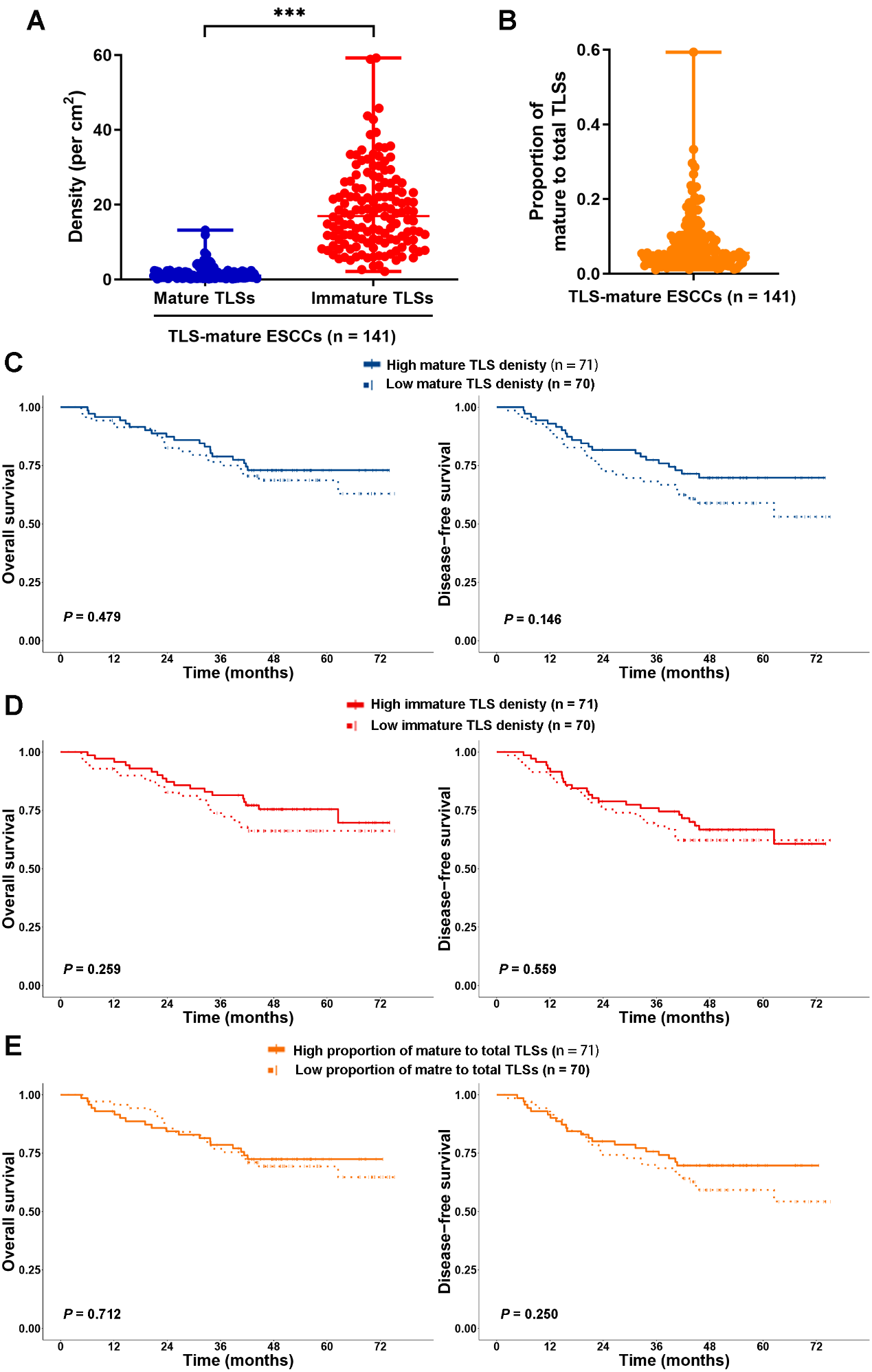
**

**Figure S2** The numbers of mature and immature TLSs and their effects on survivals in TLS-mature ESCCs. (A) Scatter plots showing the densities of mature and immature TLSs in 141 TLS-mature ESCCs from the Center B cohort. ***, *P* < 0.001 by Wilcoxon signed rank test for matched samples. (B) Scatter plot showing the proportion of mature to total TLSs in 141 TLS-mature ESCCs from the Center B cohort. (C-E) Kaplan-Meier curves for overall and disease-free survivals among 141 TLS-mature ESCCs in the Center B cohort with high (n = 71) and low (n = 70) mature TLS densities (C), immature TLS densities (D), and proportions of mature to total TLSs (E). *P* values by Kaplan-Meier analysis with log-rank test.

**
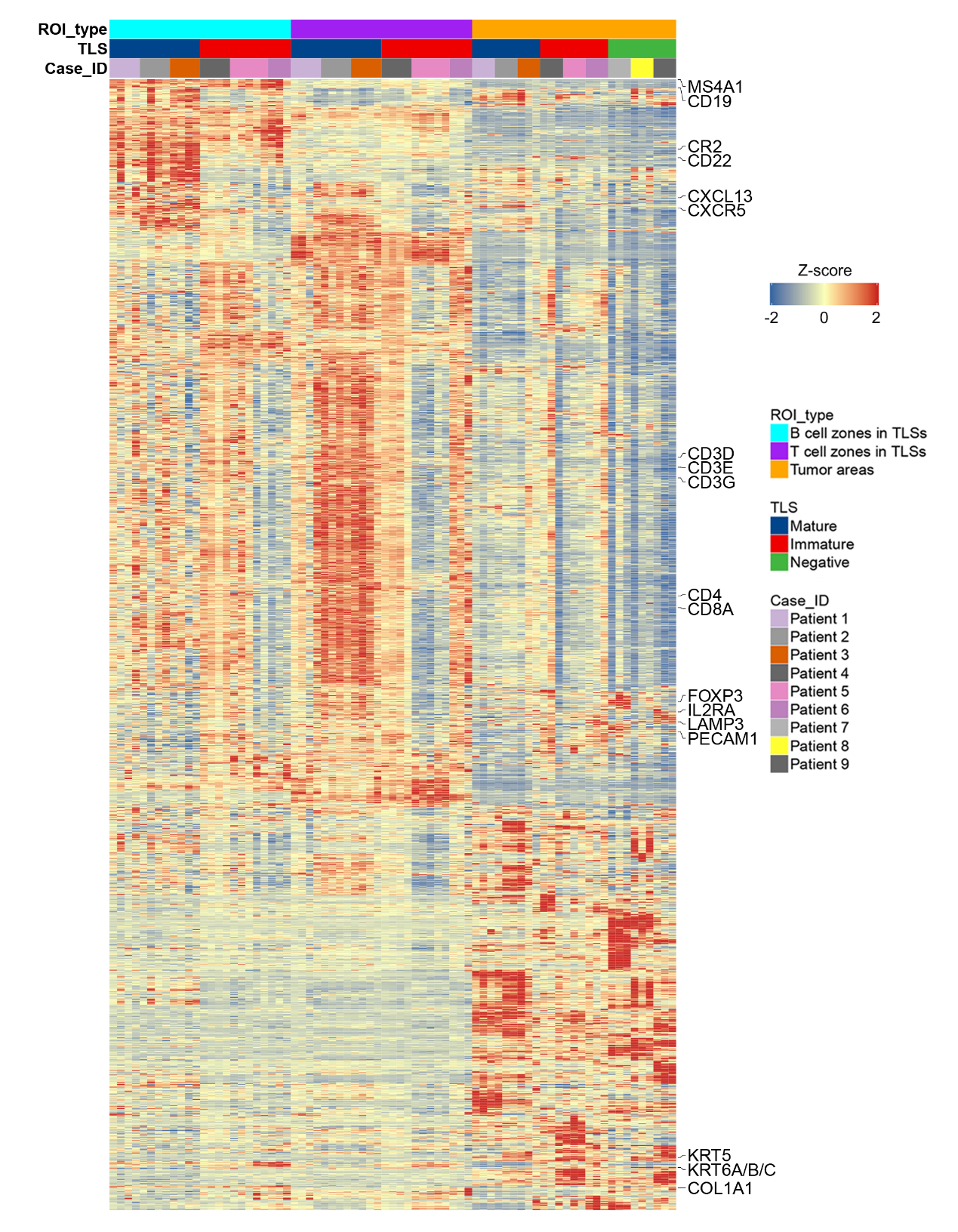
**

**Figure S3** Heatmap showing expression of genes sequenced with the Cancer Transcriptome Atlas panel in B and T cell zones of TLSs and tumor areas from TLS-mature, TLS-immature and TLS-negative ESCCs by GeoMx digital spatial profiling.


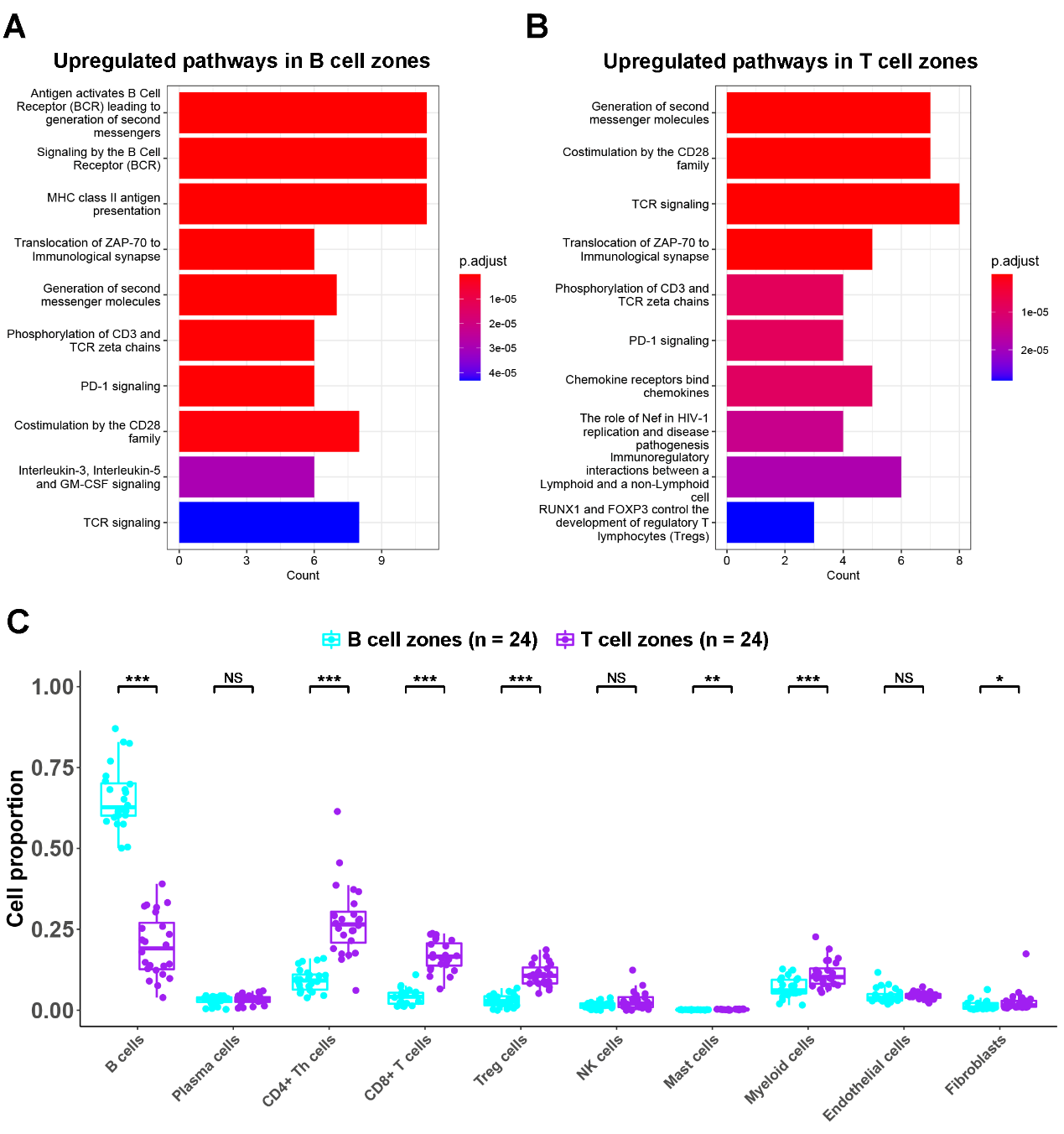


**Figure S4** Transcriptomic characteristics of B and T cell zones in TLSs. (A) Histogram plot showing the top 10 upregulated pathways in B cell zones compared to T cell zones of TLSs. (B) Histogram plot showing the top 10 upregulated pathways in T cell zones compared to B cell zones of TLSs. (C) Box plots showing the differences in cell proportions in between B and T cell zones of TLSs.


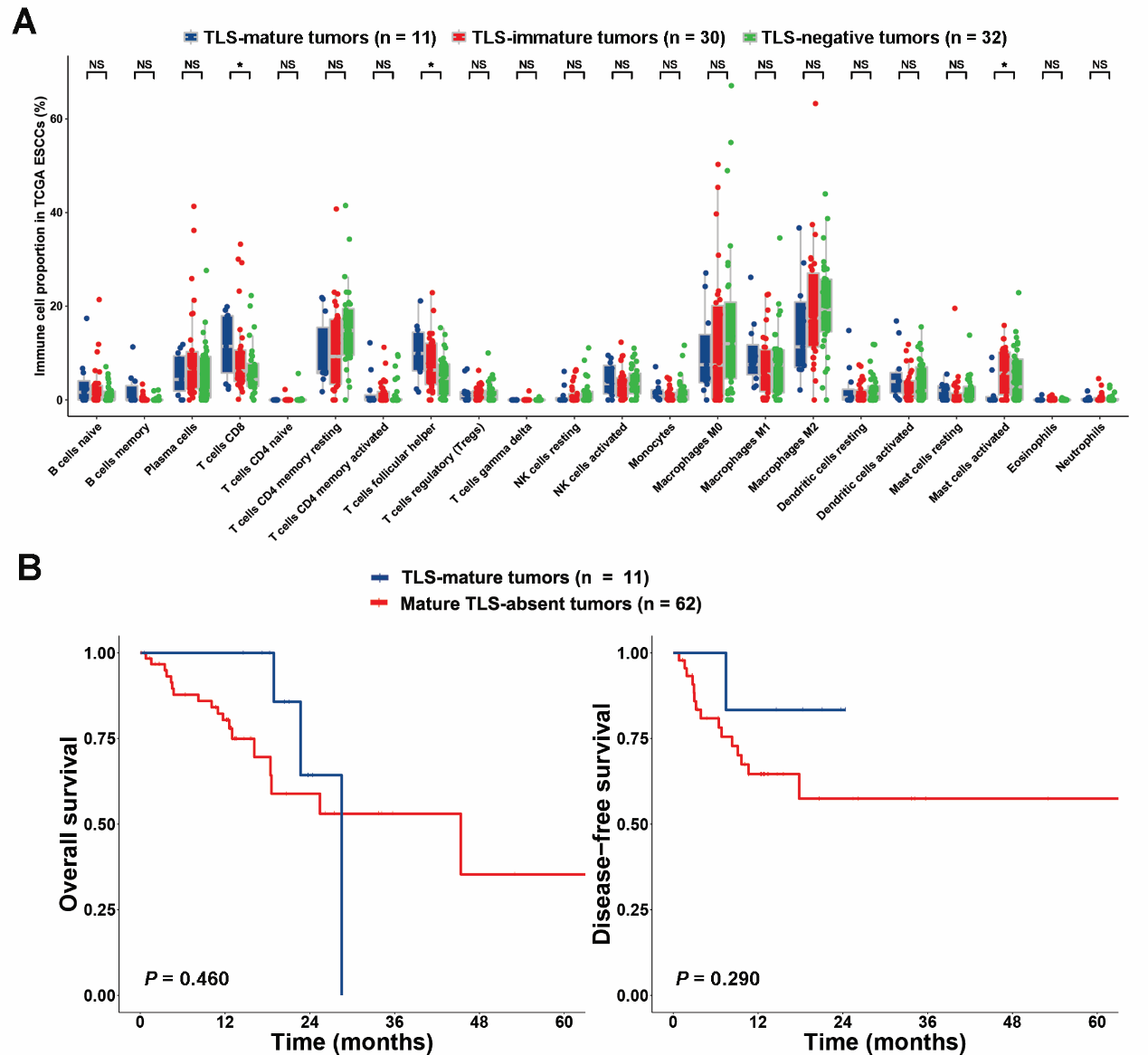


**Figure S5** Characteristics of ESCCs in TCGA cohort. (A) Box plots showing immune cell percentages estimated by CIBERSORT in TCGA TLS-mature, TLS-immature and TLS-negative ESCCs. (B) Kaplan-Meier curves for overall and disease-free survivals of TLS-mature (n = 11) and TLS-mature absent (n = 62) ESCC patients in TCGA cohort. *P* values by Kaplan-Meier analysis with log-rank test.


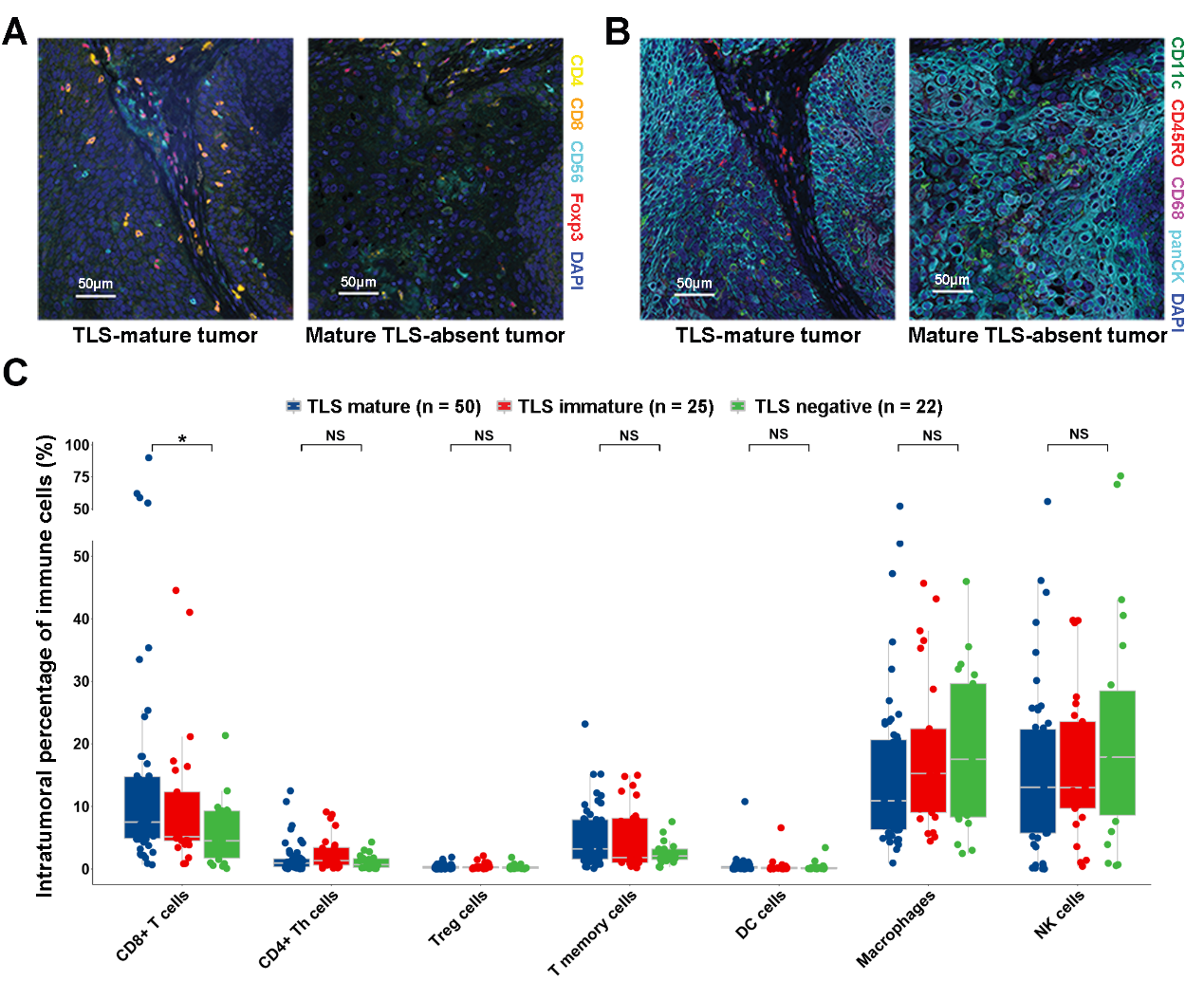


**Figure S6** Characteristics of intratumoral immune infiltration and survival of TLS-mature, TLS-immature, and TLS-negative ESCCs. (A-B) Representative mfIHC-stained images of TLS-mature and mature TLS-absent ESCCs with CD4, CD8A, CD56, FOXP3, and DAPI (A) and CD11c, CD45RO, CD68, panCK, and DAPI (B). (C) Box plots showing percentages of intratumoral immune cells between TLS-mature (n = 50), TLS-immature (n = 25), and TLS-negative (n = 22) ESCCs. *, *P* < 0.05; NS, not significant by Mann-Whitney U test with Benjamini-Hochberg correction.
