# Supplementary material for: The prognostic value and molecular properties of tertiary lymphoid structures in oesophageal squamous cell carcinoma
Source: Clin Transl Med. 2022 Oct 17;12(10):e1074. doi: 10.1002/ctm2.1074 (PMC9574489; doi:10.1002/ctm2.1074)
Supplement: Supplementary file 2 — Tables 1‐3 information [file CTM2-12-e1074-s001.docx]

**Table S1** Model performances of the deep-learning TLS classifier for TLS identification and maturation evaluation in the test set at tile-level and in the external test at slide- and patient-level

|  | Test set of 193 tiles | | External test set of 199 slides | | External set of 80 cases | |
| --- | --- | --- | --- | --- | --- | --- |
|  | TLS identification | Mature TLS identification | TLS identification | Mature TLS identification | TLS identification | Mature TLS identification |
| Accuracy | 95.3% | 97.9% | 99.0% | 96.0% | 100.0% | 97.5% |
| Precision | 100.0% | 90.0% | 99.5% | 95.9% | 100.0% | 97.6% |
| Sensitivity | 95.3% | 96.4% | 99.5% | 93.4% | 100.0% | 97.6% |
| Specificity | NA | 98.28% | 91.7% | 97. 6% | 100.0% | 97.4% |
| F1 score | 97.6% | 93.1% | 99.5% | 94.7% | 100.0% | 97.6% |

**Table S2** Univariate Cox regression analysis of association between the density of mature and immature TLSs and proportion of mature to total TLSs with overall survival and disease-free survival of 141 TLS-mature ESCC patients from the Center B cohort

| Variable | Overall survival | | | Disease-free survival | | |
| --- | --- | --- | --- | --- | --- | --- |
|  | HR | 95% CI | *P* value^†^ | HR | 95% CI | *P* value^†^ |
| Mature TLS density | 0.999 | 0.853-1.170 | 0.988 | 0.946 | 0.802-1.116 | 0.511 |
| Immature TLS density | 0.985 | 0.955-1.016 | 0.328 | 1.002 | 0.976-1.029 | 0.876 |
| Proportion of mature to total TLSs | 4.568 | 0.163-128.048 | 0.372 | 0.908 | 0.025-33.516 | 0.958 |

^†^Univariate Cox proportional hazards regression.

ESCC: esophageal squamous cell carcinoma; TLS: tertiary lymphoid structure; Center B: Cancer Hospital of Shantou University Medical College; HR: hazard ratio; CI: confidence interval.

**Table S3** ESCC primary tumors used for GeoMX digital spatial profiling analysis

| Patient | Age (years) | Gender | Tumor location | Differentiation | pT-stage | pN-stage | pM-stage | TLS status |
| --- | --- | --- | --- | --- | --- | --- | --- | --- |
| 1 | 62 | Female | Lower | Moderate | T2 | N1 | M0 | Mature |
| 2 | 71 | Male | Lower | Poor | T2 | N0 | M0 | Mature |
| 3 | 59 | Male | Lower | Moderate | T3 | N0 | M0 | Mature |
| 4 | 72 | Male | Middle | Poor | T3 | N1 | M0 | Immature |
| 5 | 62 | Male | Lower | Poor | T3 | N3 | M0 | Immature |
| 6 | 60 | Female | Middle | Moderate | T3 | N2 | M0 | Immature |
| 7 | 52 | Female | Upper | Moderate | T3 | N0 | M0 | Negative |
| 8 | 76 | Female | Middle | Well | T2 | N0 | M0 | Negative |
| 9 | 72 | Female | Middle | Moderate | T3 | N0 | M0 | Negative |

ESCC: esophageal squamous cell carcinoma.
