# Supplementary material for: The prognostic value and molecular properties of tertiary lymphoid structures in oesophageal squamous cell carcinoma
Source: Clin Transl Med. 2022 Oct 17;12(10):e1074. doi: 10.1002/ctm2.1074 (PMC9574489; doi:10.1002/ctm2.1074)
Supplement: Supplementary file 6 — Table S7 information [file CTM2-12-e1074-s004.docx]

**Table S7** Classification of sequenced genes by GeoMx

| Classification | Genes |
| --- | --- |
| Chemokines | CCL1, CCL11, CCL13, CCL14, CCL15, CCL16, CCL17, CCL18, CCL19, CCL2, CCL20, CCL21, CCL22, CCL23, CCL24, CCL25, CCL26, CCL27, CCL28, CCL3, CCL4, CCL5, CCL7, CCL8, CXCL1, CXCL10, CXCL11, CXCL12, CXCL13, CXCL14, CXCL16, CXCL2, CXCL3, CXCL5, CXCL6, CXCL8, CXCL9 |
| Major histocompatibility complex | HLA-A, HLA-B, HLA-C, HLA-DMA, HLA-DMB, HLA-DOA, HLA-DOB, HLA-DPA1, HLA-DPB1, HLA-DQA1, HLA-DQA2, HLA-DQB1, HLA-DRA, HLA-DRB1, HLA-DRB3, HLA-DRB4, HLA-DRB5, HLA-E, HLA-F, HLA-G |
| Epithelial-mesenchymal transition | FN1, ACTA2, SNAI2, COL1A1, COL1A2, COL3A1, COL5A1, COL5A1, COL6A3, LAMA3, LAMC2, ITGA2, ITGA5, ITGAV, ITGB1, FLNA, MMP1, MMP3, CD44, NT5E, PDGFRB, VCAN, TNC, SERPINH1, THBS1, TNFRSF12A, TPM1, VEGFC |
